# WHO/UNEP global surveys of PCDDs, PCDFs, PCBs and DDTs in human milk and benefit–risk evaluation of breastfeeding

**DOI:** 10.1007/s00204-016-1802-z

**Published:** 2016-07-20

**Authors:** Martin van den Berg, Karin Kypke, Alexander Kotz, Angelika Tritscher, Seoung Yong Lee, Katarina Magulova, Heidelore Fiedler, Rainer Malisch

**Affiliations:** 1 Institute for Risk Assessment Sciences (IRAS), Utrecht University, P.O. Box 80177, 3508 TD Utrecht, The Netherlands; 2 WHO/UNEP Reference Laboratory, State Institute for Chemical and Veterinary Analysis of Food (CVUA), Bissierstrasse 5, 79114 Freiburg, Germany; 3 Department of Food Safety and Zoonoses, World Health Organization, Ave Appia 20, 1211 Geneva 27, Switzerland; 4 Stockholm Convention Secretariat, United Nations Environment Programme (UNEP), International Environment House, 1219 Châtelaine, Geneva, Switzerland; 5 Division of Technology, Industry and Economics/Chemical Branch, United Nations Environment Programme (UNEP), Chemin des Anémones 11-13, 1219 Châtelaine, Geneva, Switzerland

**Keywords:** Human milk, Dioxins, PCBs, DDT, Breastfeeding, Benefit–risk

## Abstract

Since 1987, the World Health Organization (WHO) carried out global surveys on polychlorinated dibenzo-*p*-dioxins (PCDDs), polychlorinated dibenzofurans (PCDFs) and polychlorinated biphenyls (PCBs) in human milk. This study presents a review of the three most recent surveys from 2000 to 2010, including DDT. The objective was to identify global quantitative differences and provide baseline information for 52 countries or provide time-trends for countries with previous data. Individual human milk samples were collected following a WHO-designed procedure and combined to form a national pooled sample. Here, we report global levels for PCDDs, PCDFs, PCBs and the sum of *o*,*p*′-DDT, *p*,*p*′-DDT, *o*,*p*′-DDE, *p*,*p*′-DDE, *o*,*p*′-DDD and *p*,*p*′-DDD (**Σ**DDTs). A concise risk–benefit evaluation related to human milk contamination with these persistent organic pollutants (POPs) was also done. Large global and regional differences were observed. Levels of PCDDs and PCDFs were highest in India and some European and African countries. PCB levels were highest in East and West Europe. The highest levels of **Σ**DDTs were found in less industrialized countries. A temporal downward trend for PCDDs, PCDFs and PCBs is indicated. A risk–benefit assessment indicates that human milk levels of PCDDs, PCDFs and PCBs are still significantly above those considered toxicologically safe, while **Σ**DDTs are below or around those considered safe. With respect to potential adverse health effects, a more dominant role of in utero exposure versus lactational exposure is indicated. If potential adverse effects are balanced against positive health aspects for (breastfed) infants, the advantages of breastfeeding far outweigh the possible disadvantages. Our observations provide a strong argument to plea for further global source-directed measures to reduce human exposure further to dioxin-like compounds.

## Introduction

Since the mid-eighties, the World Health Organization (WHO) coordinates a comprehensive global monitoring program on polychlorinated biphenyls (PCBs), polychlorinated dibenzo-*p*-dioxins (PCDDs) and polychlorinated dibenzofurans (PCDFs) (Malisch et al. 2010; Malisch and Van Leeuwen 2003; WHO 1989, 1996). This program focuses particularly on the exposure of breastfed and aims to identify the impact of measures taken to reduce or prevent environmental exposure to these chemicals.

The Stockholm Convention on Persistent Organic Pollutants (POPs) has the objective to protect human health and environment from POPs by reducing or eliminating their releases into the environment. Under this convention, human milk was recommended as a core matrix for biomonitoring. To evaluate the effectiveness of the Stockholm Convention, the results from these human milk surveys will be used as a baseline to identify possible global temporal trends and spatial distributions of POPs. These surveys also provide a suitable basis for possible source-directed reduction measures of POPs in human food and milk on a country-by-country basis.

For biomonitoring of POPs, human milk is the best matrix to use, because it is easily available, collecting is non invasive, and its high lipid content makes the extraction of POPs easy (Liem et al. 2000). PCDDs, PCDFs and PCBs in human milk are also a good reflection of the body burden, as human blood and adipose tissue concentrations are often (closely) similar (Needham et al. 2011; Todaka et al. 2010).

Experimental and epidemiological studies indicate that the early life stage is most sensitive for the effects of dioxin-like (DL) compounds and PCBs (Birnbaum and Tuomisto 2000; Peterson et al. 1993). Therefore, placental transfer and uptake via human milk can be of toxicological significance. Consequently, the exposure of breastfed infants is a challenge for the benefit–risk assessment of human milk, because significant health benefits concur with possible adverse health effects of these POPs (Aliyu et al. 2010; Landrigan et al. 2002; Mead 2008).

This article reviews three global surveys of PCDDs, PCDFs, PCBs and DDT in human milk. These surveys are meant to identify worldwide differences of human milk contamination, but not meant to derive a “ranking” of countries with respect to risk assessment for the breastfed infant. Based on the global levels observed during these surveys, a concise benefit**–**risk evaluation is included for the breastfed infant.

## Analytical aspects

The State Institute for Chemical and Veterinary Analysis of Food (Freiburg, Germany) analyzed the composite human milk samples from various countries following the earlier WHO-designed procedure (http://www.who.int/foodsafety/chem/POPprotocol.pdf) (WHO 2007).

### Protocols

In short, samples were collected following a protocol dealing primarily with number and type of samples, donor selection, storage, pooling and shipping of samples. Inclusion criteria for donating mothers were: (a) primiparae, (b) healthy, (c) exclusive breastfeeding to one child and (d) local resident for about 5 years. Furthermore, WHO requirements in conducting studies with human milk are: (a) breastfeeding should always be promoted and supported, (b) sampling should neither be an unnecessary burden to the mother nor compromise the nutritional status of the infant, (c) samples should be as representative of the relevant population of the country as possible, (d) donor selection should take into account possible contributing factors, e.g., dietary habits and demographic differences. Approval from all national ethics committee was obtained.

The third survey (2000–2003) included pooled milk samples from at least two well-defined groups of ten mothers. If possible, a high- and low-exposure group was selected. If more than one pooled sample was donated, the median value was used for comparison.

For the fourth (2005–2007) and fifth (2008–2010) surveys, pooled milk samples from at least 50 donors per country were used. The donors were considered representative for a general background situation in a specific country. The samples of individual mothers remain available for future analysis of other POPs.

**Fig. 1:**
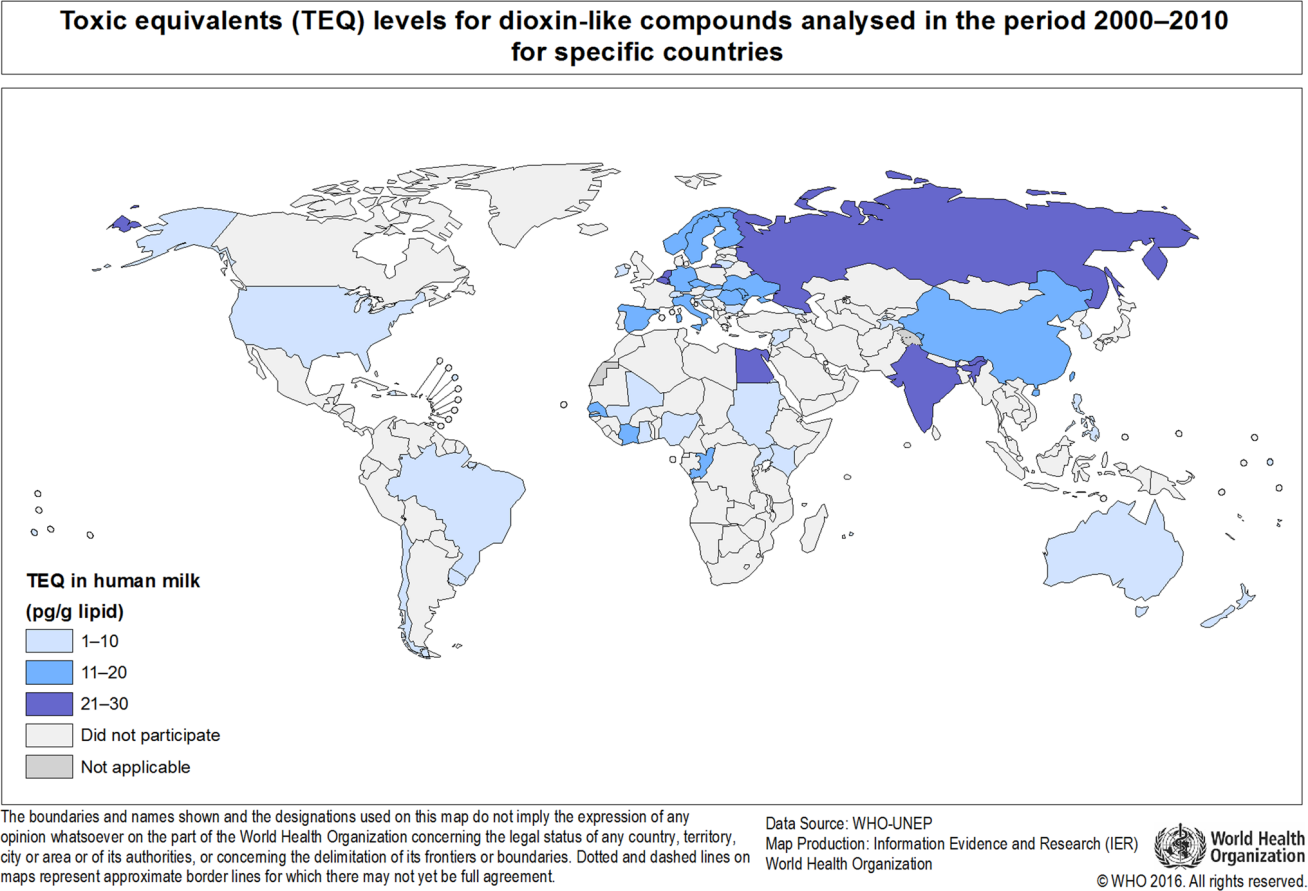
Participating countries in the WHO/UNEP human milk global surveys and levels of dioxin-like compounds expressed in TEQs (Van den Berg et al. 2006)

**Fig. 2:**
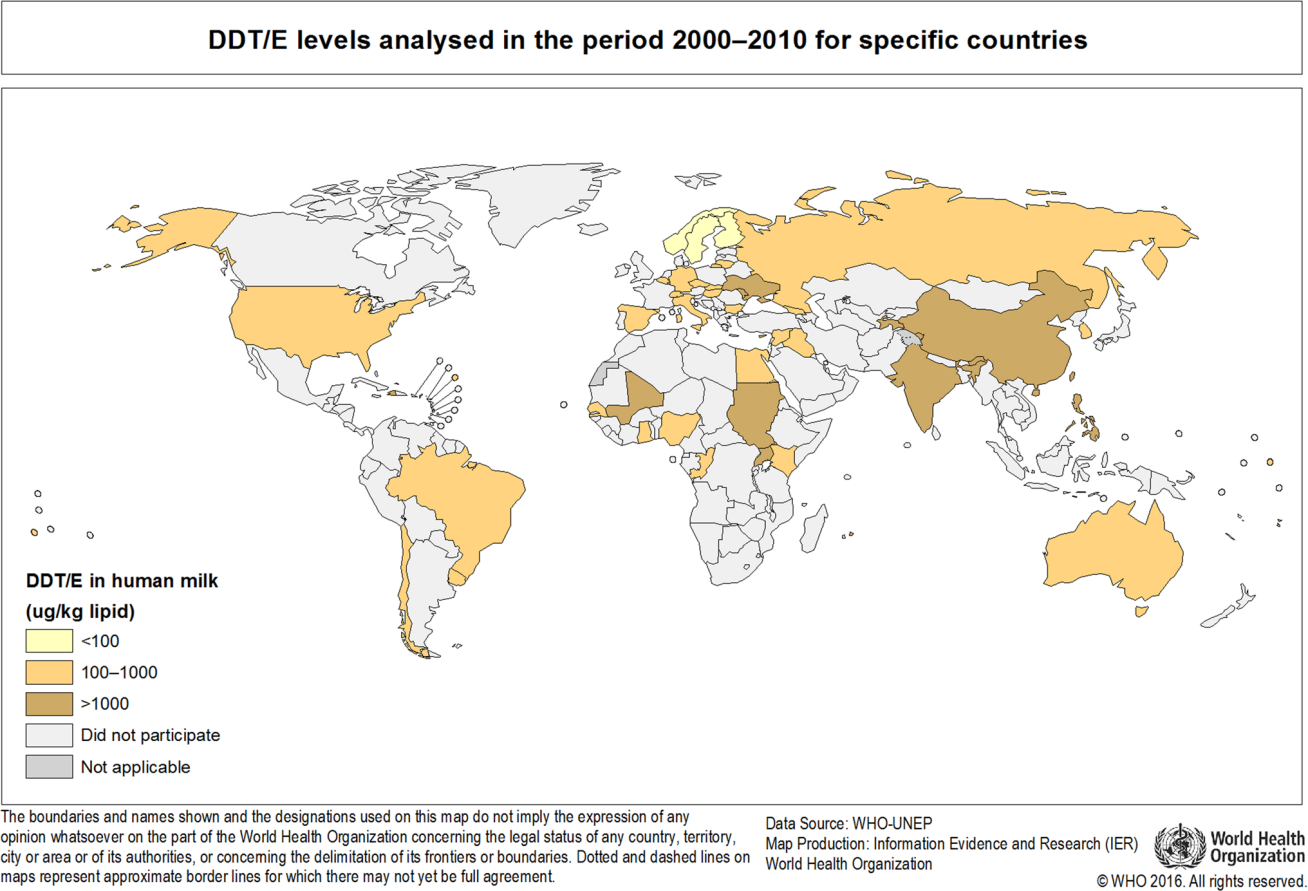
Participating countries in the WHO/UNEP human milk global surveys and levels of **Σ**DDTs

**Fig. 3:**
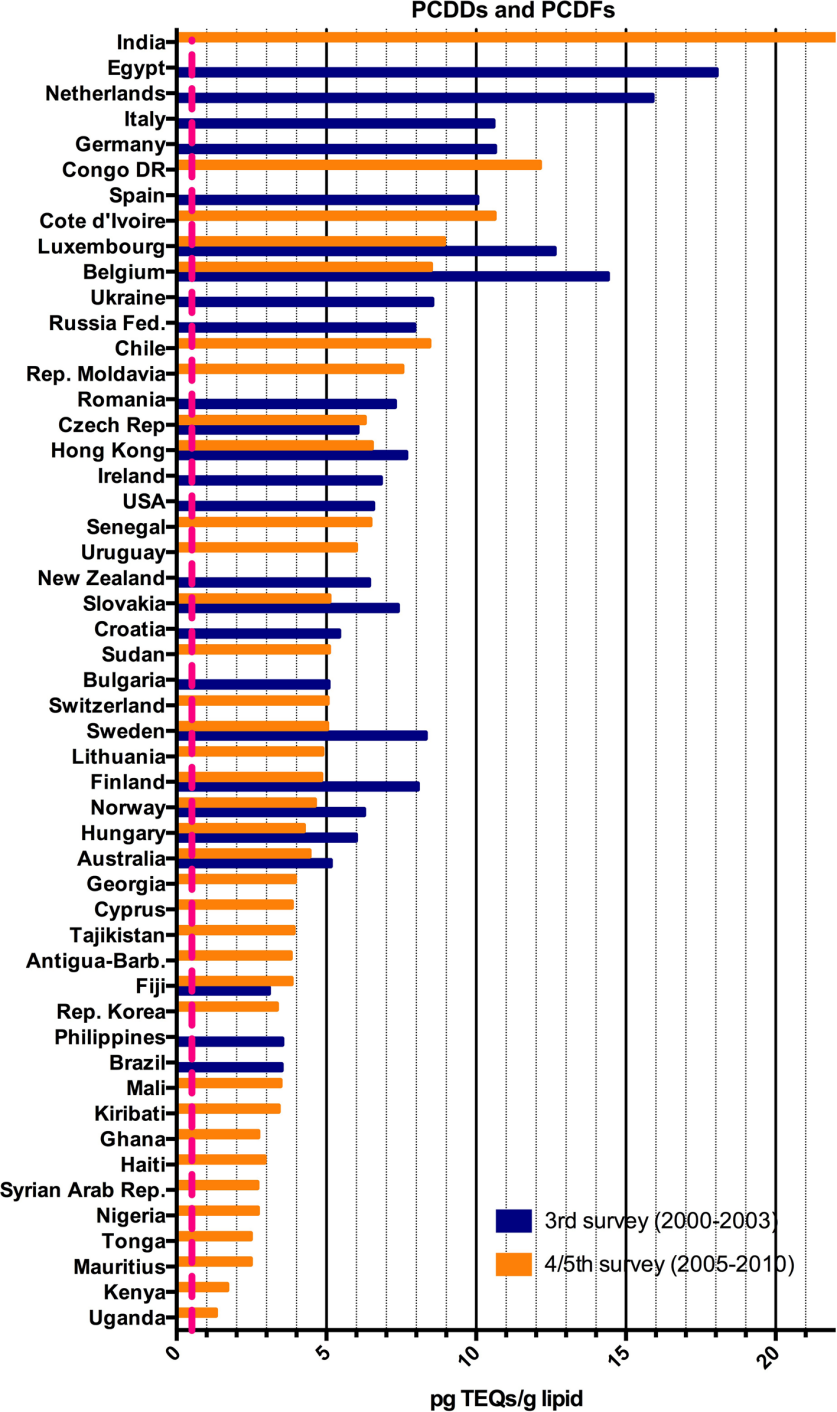
Results of the WHO/UNEP surveys for PCDDs and PCDFs in TEQs (pg/g lipid) in pooled human milk samples from different countries. The *dotted red line* represents the calculated safe level of these compounds for the breastfed infant (color figure online)

**Fig. 4:**
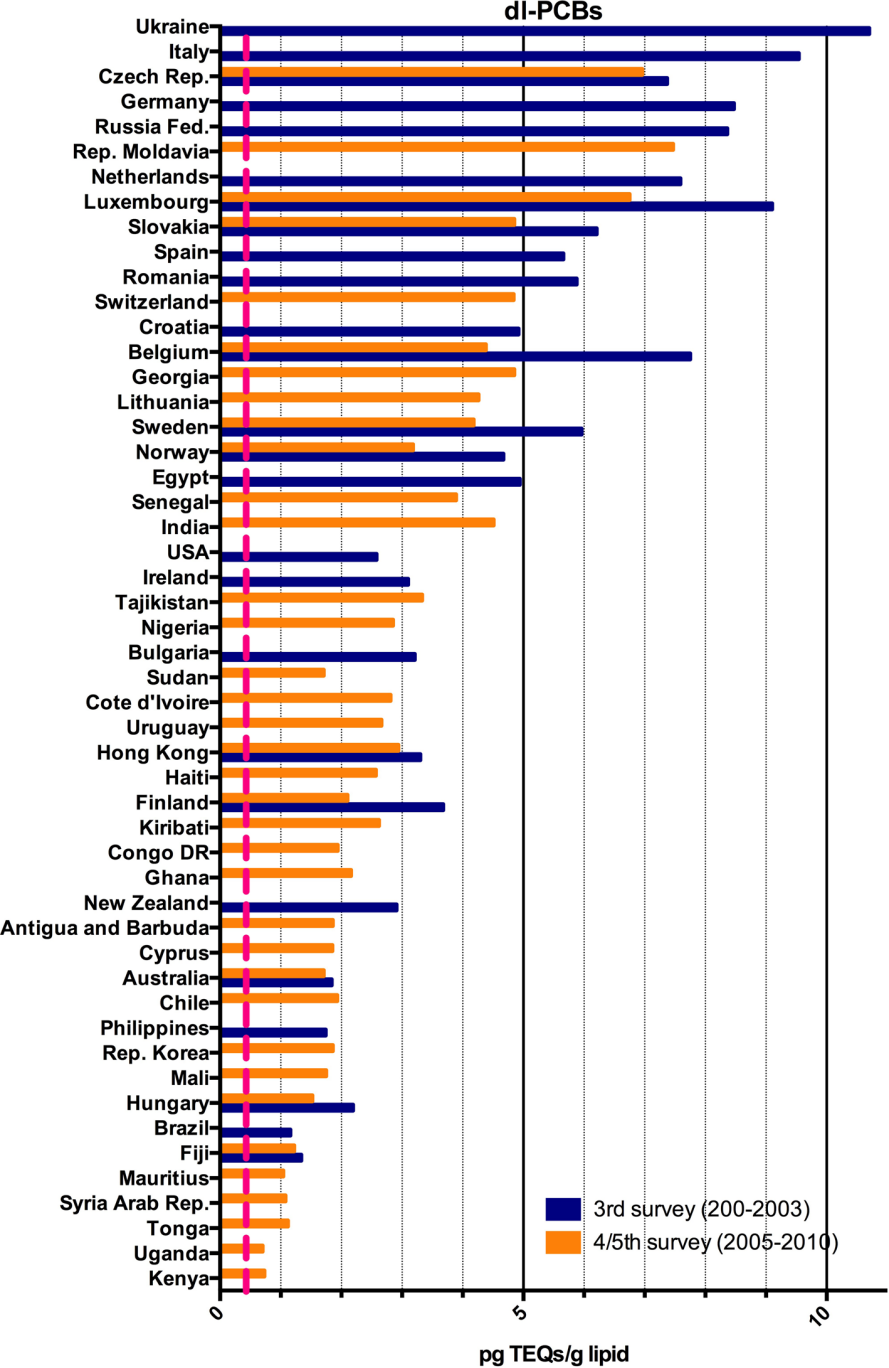
Results of the WHO/UNEP surveys for DL-PCBs in TEQs (pg/g lipid) in pooled human milk samples from different countries. The *dotted red line* represents the calculated safe level of these compounds for the breastfed infant (color figure online)

**Fig. 5:**
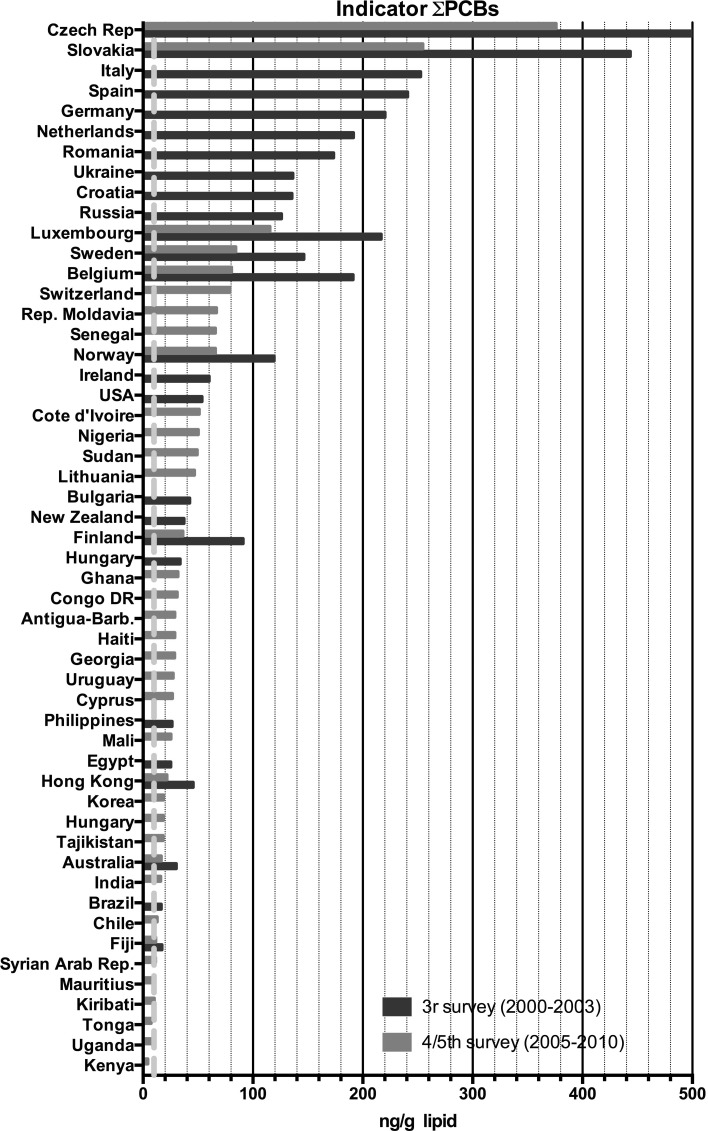
Results of the WHO/UNEP surveys for the sum of the indicator PCBs in ng/g lipid in pooled human milk samples from different countries. The *dotted red line* represents the calculated safe level of these compounds for the breastfed infant (color figure online)

**Fig. 6:**
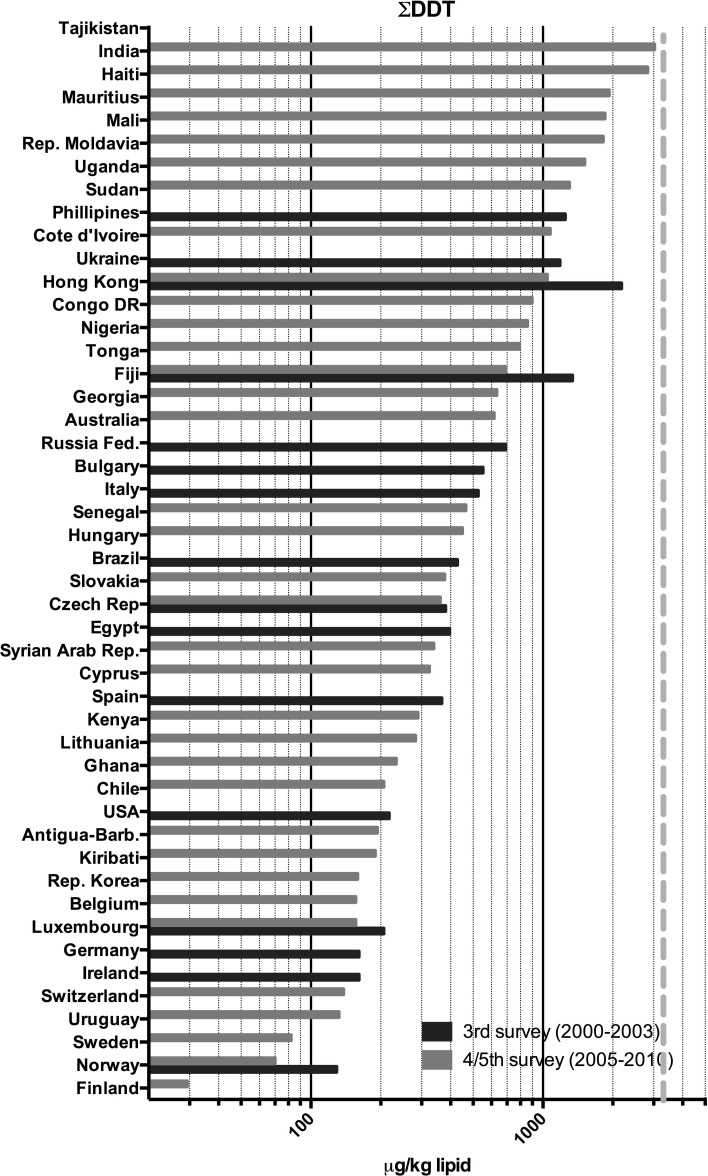
Results of the WHO/UNEP surveys for the sum of DDT-like compounds in μg/kg lipid in pooled human milk samples from different countries. The *dotted red line* represents the calculated safe level of these compounds for the breastfed infant (color figure online)

In the third survey, 102 samples were analyzed for PCDDs, PCDFs, PCBs and partly for DDT. In the fourth and fifth survey 13 and 23 countries sent in, respectively, 16 and 28 samples. As the fourth and fifth surveys were done within a relatively short time period, it was decided to combine the data in this study. In Figs. 1 and 2, the participating countries for dioxin-like compounds and **Σ**DDTs are presented, while in Figs. 3, 4, 5 and 6, more detailed analytical data are presented for each country.

### Chemical analysis

The analytical procedure for PCDDs, PCDFs and DL-PCBs has been described earlier (Malisch et al. 2000; Malisch and Dilara 2007; Malisch and Van Leeuwen 2002). The limit of quantification (LOQ) expressed as WHO toxic equivalencies (TEQs) was ~0.1 pg/g lipid. Toxic equivalency factors (TEFs) for DL compounds as proposed by the 2005 WHO expert meeting have been used (Van den Berg et al. 2006). The LOQ for individual non DL-PCBs was <0.05 ng/g lipid and for DDT, DDD and DDE ~0.5 ng/g lipid. Concentrations of the indicator PCBs 28, 52, 101, 138, 153 and 180 are expressed as the sum of these PCBs (ΣPCBs). The *mono*-*ortho* substituted PCB 118 has DL properties and included in the TEQ calculations.

The chemical analysis of DDT and its metabolites has been described earlier (Hedley et al. 2010; UNEP 2007). **Σ**DDTs concentrations were calculated after correction for molecular weight to provide the sum of *o*,*p*′-DDT, *p*,*p*′-DDT, *o*,*p*′-DDE, *p*,*p*′-DDE, *o*,*p*′-DDD and *p*,*p*′-DDD.

## Global occurrence

### PCDDs, PCDFs and PCBs

#### Third survey

**Fig. 7:**
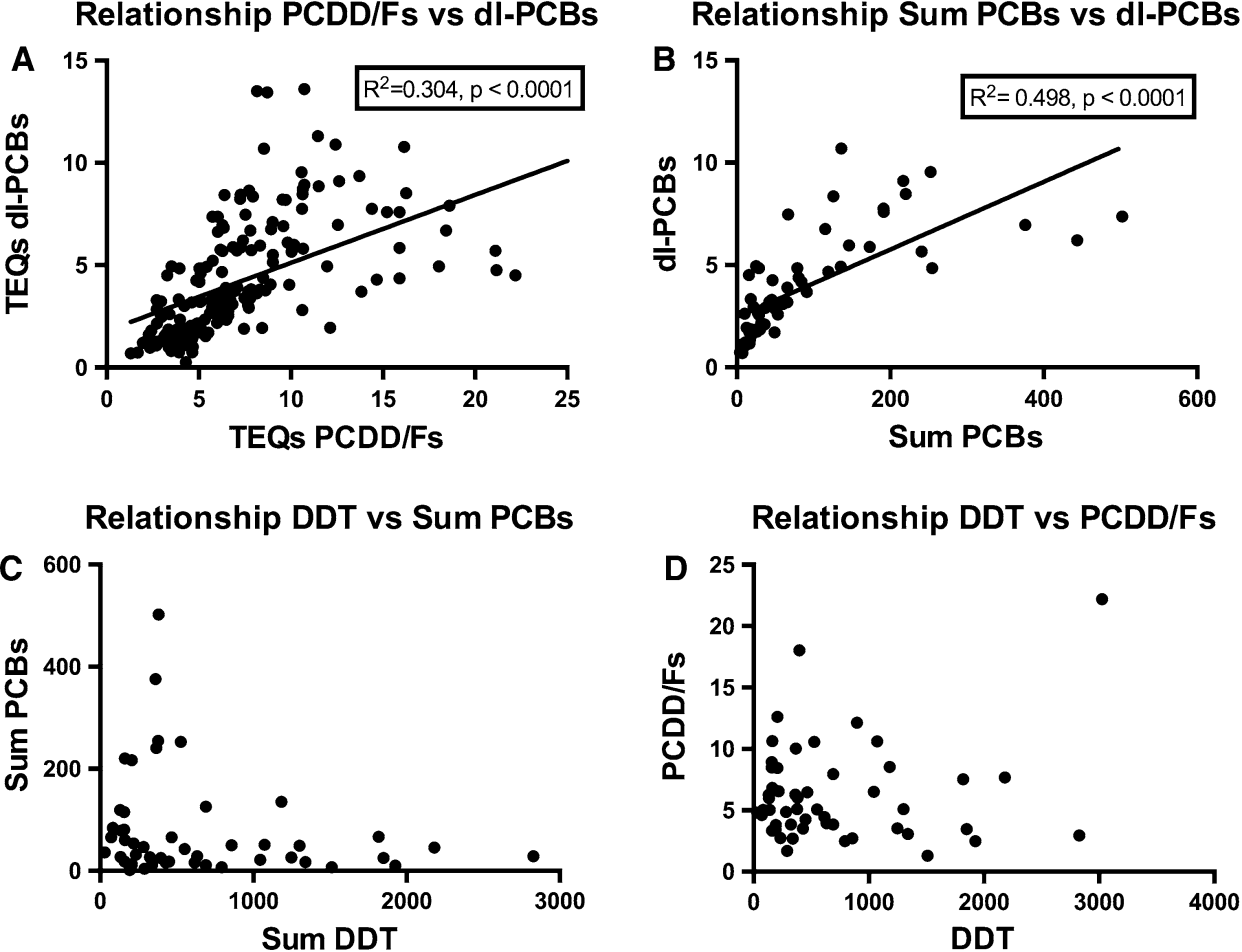
Relationships between PCDD, PCDF, PCB and DDT levels in pooled human milk samples from the WHO/UNEP surveys

The results for the third survey (2000–2003) are shown in Figs. 3, 4, 5 and 6. Between countries, approximately one order of magnitude variation was found. The lowest concentrations of dioxin-like compounds were predominantly found in less industrialized countries (Fig. 1). Comparatively higher levels were found in the more highly industrialized European countries. Many Western and Eastern European countries had comparatively high levels of DL-PCBs, with the highest observed in the Ukraine. Remarkably high levels of PCDDs and PCDFs were found in Egypt. In contrast, the levels of PCBs in Egypt were much lower compared to many other industrialized countries studied (Figs. 3, 4, 5). An almost linear relationship was found between PCDDs and PCDFs versus DL-PCBs that becomes less distinct at higher concentrations (Fig. 7a). For most industrialized countries, the contribution of PCDDs/PCDFs and DL-PCBs was approximately equal. The levels of indicator PCBs measured in the third survey vary widely between countries. In Europe, high levels were found in, e.g., the Czech Republic and Slovakia. Significantly lower levels were observed in the Southern Hemisphere (Fig. 5). As could be expected, a strong relationship between DL-PCBs and indicator ΣPCBs was found (Fig. 7b).

#### Fourth and fifth surveys

The results of the combined fourth and fifth surveys (2005–2010) expressed in TEQ or ΣPCBs are also shown in Figs. 3, 4, 5 and 6. Many countries provided only one pooled sample, and no median levels could be established. PCDD, PCDF and PCB levels were again by far the highest in Europe. The levels in less industrialized countries were significantly lower than those in Europe. This survey period confirms again that many countries in the Southern Hemisphere have low levels of PCDDs, PCDFs and PCBs (Figs. 3, 4, 5). In Africa, the widest variation in contamination was observed. Kenya and Uganda had the lowest levels of PCDDs and PCDFs, while Côte d’Ivoire and the Democratic Republic of the Congo had the highest levels in these surveys (Fig. 3). It is noticeable that the latter countries did not have exceptional high levels of PCBs (Figs. 4, 5), indicating that the high PCDDs and PCDFs contamination is not associated with PCB-related occurrence. Most Asian countries studied in these surveys had relatively low levels of PCDDs/PCDFs and PCBs. However, exceptional high levels of PCDDs and PCDFs were detected in India (Fig. 2), but levels of PCBs in this country were not high (Figs. 4, 5). Limited information was obtained from the Caribbean and Central/South American countries, but relatively low levels of PCDDs, PCDFs and PCBs were found for those countries studied. Similar, levels in Australia were low, indicating limited contamination of human milk with these compounds (see Fig. 1).

**Fig. 8:**
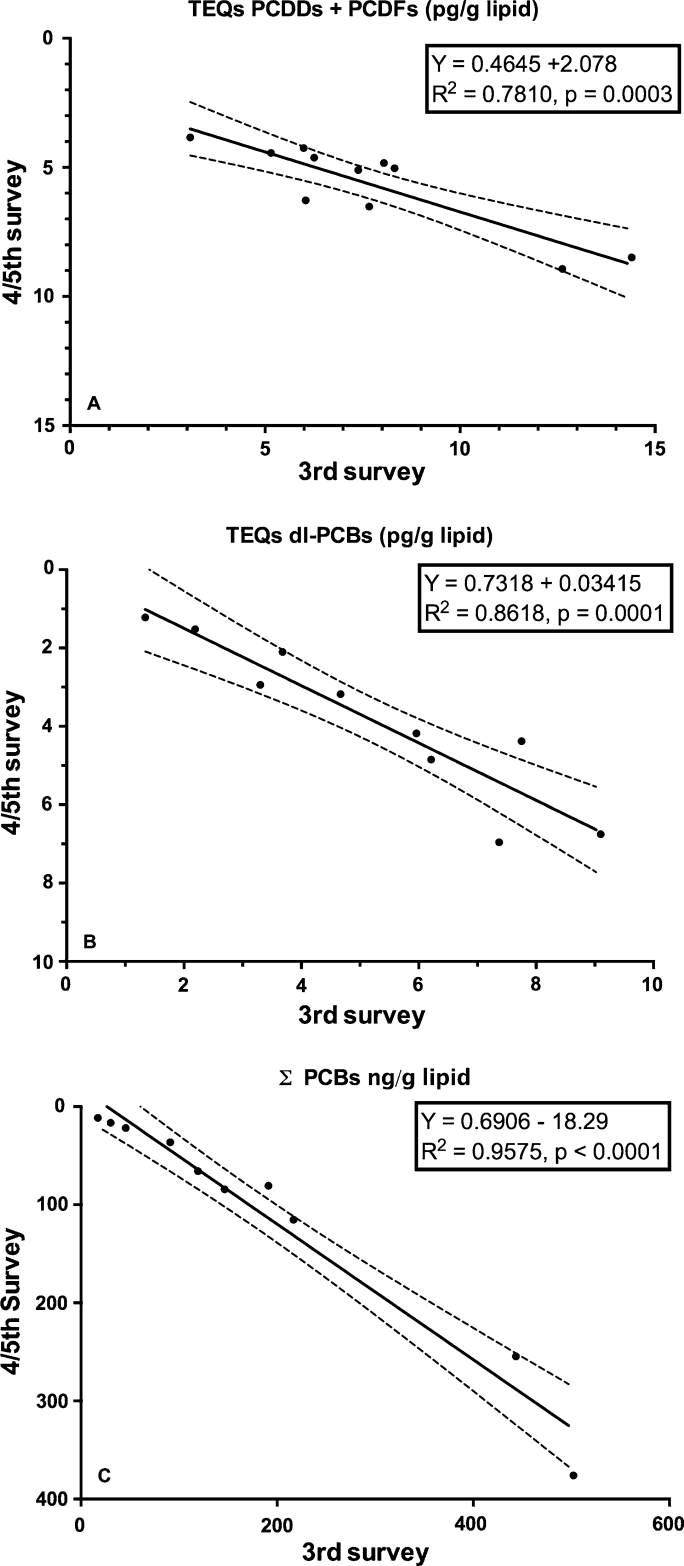
Decline rates of PCDD, PCDF and PCB in some selected countries that have been studied during subsequent WHO/UNEP human milk survey periods (2000–2003 and 2005–2010)

To detect a possible downward temporal trend of PCDDs, PCDFs and PCBs, a country-by-country comparison was made for those countries that participated in both survey periods (Fig. 8a–c). Although the number of countries that could be compared is limited, these results may indicate that PCDD, PCDF and PCB levels in human milk further declined in the first decade of the twenty-first century.

## DDT and metabolites

The highest ΣDDTs levels were found in a number of Asian countries and Haiti (Figs. 2, 6). The high ΣDDTs levels in Hong Kong SAR in the third survey are noticeable, but a significant reduction was observed in the fifth survey. Surprisingly, ΣDDTs levels in Tajikistan were nearly a factor of three higher than in India. If the ranking order of ΣDDTs levels is reviewed, the tropical countries represent the majority of the upper half of ΣDDTs human milk levels. This observation certainly reflects its use in relation to the occurrence and prevention of malaria in these regions in recent times. In addition, it should be noted that no relationship was found between PCDDs, PCDFs and PCBs versus ΣDDTs levels, which illustrates the country-by-country differences in industrialization versus (former) malaria prevention with DDT (Fig. 7c, d).

## Discussion

### Global levels

This study is the first in its kind reviewing global temporal and spatial contamination of PCDDs, PCDFs, PCBs and DDT of human milk. For many developing countries, this is the first time that such data are evaluated in a global perspective. Our results show that geographical areas with the highest levels are mostly associated with industrialization (Figs. 1, 2). In this review, the results of the fourth and fifth survey were combined. With approximately 5 years in between, we expected no major effect on the overall conclusions, because of the high environmental persistence and slowly decreasing levels of these POPs. Many European countries were found in the upper half (>5 pg TEQs/g lipid) of the human milk levels of PCDDs and PCDFs (Fig. 3). Surprisingly, some less industrialized countries from Asia, Africa and South America had similar high levels. In contrast, PCB levels in these non-European countries were relatively low, <100 ng/g lipid (Figs. 4, 5). Thus, PCB contamination contributes little to the high levels of TEQs in these countries. Combustion and industrial processes more likely contribute to the human exposure of PCDDs and PCDFs (UNEP 2011). Clay consumption (geophagia) by pregnant women is the likely source of PCDD and PCDF exposure in some African countries that can lead to high levels in human milk (Reeuwijk et al. 2013). A downward trend appears to be present for DL compounds and PCBs during our two survey periods (Fig. 8a–c). A reduction in environmental levels during our surveys can be one explanation. However, the lower number of European countries in the fourth and fifth surveys may also be responsible or contribute to this apparent decrease. To resolve this issue, time-trends should best be followed on country-by-country basis. This has already been done for, e.g., Germany, Norway, The Netherlands, Japan and the USA, and confirms a downward trend (Furst et al. 1994; LaKind et al. 2001; Liem et al. 2000; Papke 1998). This suggests that the possible downward trend in the WHO surveys may indeed be real and no coincidence due to the pooled samples. The global distribution for ΣDDTs is almost exclusively associated with countries where malaria is still a significant health problem (Fig. 2).

### Risks

There is an ongoing discussion regarding the benefits *versus* risks of breastfeeding in relation to the presence of POPs in human milk. The WHO has always promoted exclusive breastfeeding because of its positive health aspects for the neonate and mother, but pros and con’s remain. The results of this WHO/UNEP study can also be used to weight the benefits *versus* risks on a global level.

The WHO derived a Tolerable Daily and (Provisional) Monthly Intake (TDI or PTMI) for DL equivalents (TEQ) of 1–4 and 70 pg TEQ/kg bw per day or month, respectively (WHO 2000, 2002). The US-EPA proposed an oral reference dose (RfD) of 0.7 pg TCDD/kg bw per day (US-EPA 2010), and the ATSDR set a minimal risk level (MRL) for acute and semi-chronic exposure of 20 and 1 pg TCDD/kg bw per day, respectively (ATSDR 1998). By default, these safety standards are meant for chronic life time exposure and not applicable for the breastfeeding situation, which covers a much shorter time period of life. However, in-depth analysis of the underlying data shows that the most sensitive endpoints used to derive these safety standards often involve pre- or postnatal experimental studies. These effects include immune suppression, sperm count, genital malformation in rodents, while neurobehavioral effects are among the most sensitive endpoints in non-human primates (WHO 2000, 2002). Thus, scientifically sound arguments can be given to apply these TDI or PTMI values also for breastfed infant (Table 1).

**Table 1:** Different safety standards for dioxin-like compounds (TEQ) and DDT with calculated equivalent human milk levels

Organization	Safety standard		Equivalent milk level	Endpoint
		PCDD/PCDF/PCB (TEQ)	PCDD/PCDF/PCB (TEQ)	
WHO (2000)	TDI	1–4 pg/kg bw day	0.2**–**0.9 pg/g lipid	Perinatal effects rodents and monkeys
US-EPA (2010)	RfD (proposed)	0.7 pg/kg bw day	0.2 pg/g lipid	Postnatal/childhood exposure humans
ATSDR (1998)	MRL subchronic	1 pg/kg bw day	0.2 pg/g lipid	Postnatal effect monkeys

		Total PCBs	Total PCBs	
ATSDR (2004)	MRL subchronic	0.03 μg/kg bw day	7 ng/g lipid	Postnatal effect monkeys

		DDT	DDT	
WHO (2001)	TDI	10 μg/kg bw day	2300 ng/g lipid	Developmental toxicity in rats

Information and formula used

Lipid set in human milk = 3.5 % and consumption 125 g milk/kg bw day

$$\left[ {{\text{Acceptable}}\;{\text{concentration}}} \right] = \frac{{[{\text{Safety}}\;{\text{standard}}({\text{pg}}\;{\text{or}}\;\upmu{\text{g}}/{\text{kg}}\;{\text{bw }}\;{\text{day}})]}}{{4.375\;{\text{g}}\;{\text{lipid}}/{\text{kg}}\;{\text{bw}}\;{\text{day}}}}$$

Recently, the uptake via breastfeeding was estimated from 30 to more than 200 pg TEQ/kg bw per day (Li et al. 2009) and our results are in line with these data. Therefore, it can be concluded that the WHO safety standards for DL compounds are exceeded with one to two orders of magnitude during a period from several months to more than a year for the breastfed infant from all countries studied in these surveys (Figs. 3, 4). For PCBs as a group, the ATSDR minimum risk level (MRL) is 0.03 μg/kg bw per day (ATSDR 2000), and again our review indicates that this safety standard is exceeded by one to two orders of magnitude for the countries included in these surveys (Fig. 5). Thus, in all countries studied, the levels in TEQs of DL compounds and ΣPCBs in human milk and its associated maternal body burden are still one to two orders of magnitude above those considered toxicologically safe in early childhood.

Safety standards values have also been derived for DDT, and a provisional TDI of 10 μg/kg per day by the WHO was based on developmental toxicity in the rat (WHO 2001). The US-EPA and ATSDR both derived a RfD and MRL of 0.5 μg/kg bw per day for acute and intermediate oral exposure situations (ATSDR 2004; US-EPA 2011). The levels of ΣDDTs are below or around those considered to be safe in the countries studied during these surveys (Fig. 6) and from a toxicological point may be considered as acceptable in human milk.

Several epidemiological studies have focused on the risks of pre- and postnatal exposure to DL compounds, and effects on thyroid hormones, immunology, psychomotor and physical development have been observed in the breastfed infant. However, these effects were often transient and their clinical relevance discussed (Koopman-Esseboom et al. 1994, 1996; Pluim et al. 1993; Weisglas-Kuperus et al. 1995, 2000, 2004). Very recently, a study from Spain and Greece reported a negative relationship between maternal levels of DL compounds and anogenital distance in newborns (Vafeiadi et al. 2013). Many of these studies could not differentiate between possible effects of DL compounds (in TEQ) or PCBs due to their simultaneous occurrence in human milk. However, several studies provide evidence that prenatal exposure is more important than breastfeeding, which is in line with results from experimental studies with rodents and monkeys (Peterson et al. 1993). Taken together, it must be concluded that over the last two decades maternal body burdens of DL compounds and/or PCBs in industrialized countries, reflected in human milk levels, were still sufficiently high to cause subtle effects in the neonatal and early childhood period.

Human health effects of DDT and its metabolites have also been extensively studied, and adverse health effects in the neonatal period or early childhood have been implicated (Eskenazi et al. 2009; WHO 2011). Again differentiation between effects due to in utero versus neonatal exposure is often not possible. Effects on thyroid hormones or growth in childhood or puberty have been reported, but results are not equivocal (Alvarez-Pedrerol et al. 2008; Asawasinsopon et al. 2006; Chevrier et al. 2008; Gladen et al. 2004; Karmaus et al. 2002; Rogan et al. 1987; Takser et al. 2005). These effects of DDT on thyroid hormones and body growth may well be transient, small and possibly not clinically relevant (Feeley and Brouwer 2000; Rogan et al. 1987).

More significant effects of DDT and/or DDE have been observed on neurocognitive and behavioral development that may persist into childhood (Darvill et al. 2000; Eskenazi et al. 2006; Gladen and Rogan 1991; Gladen et al. 1988; Pan et al. 2010; Ribas-Fito et al. 2006; Rogan et al. 1986; Torres-Sanchez et al. 2007). These effects are not consistent, but most indicate that the maternal body burden and placental transfer are more important than breastfeeding itself (Aliyu et al. 2010; Mead 2008). In addition, conflicting results have been obtained with respect to the transient nature of these cognitive developmental effects, but a prolonged effect into childhood cannot be excluded. In relation to this reduced cognitive or neurobehavioral development in breastfed infants, it must be recognized that experimental studies provide supporting evidence for such an effect (Arendt 2008).

Possible effects of DDT and/or DDE on the immune system have also been reported, but results are still inconclusive (Dallaire et al. 2004; Dewailly et al. 2000; Park et al. 2008; Sunyer et al. 2005). One study found a positive relationship between prenatal DDE exposure and infection rate, but a confounding role of DL compounds could not be excluded (Dallaire et al. 2004).

 Possible anti-androgenic effects after perinatal exposure to DDT and/or DDE in relation to hypospadias, anogenital distance and chryptochordism have also been studied (Bhatia et al. 2005; Brucker-Davis et al. 2008; Damgaard et al. 2006; Flores-Luevano et al. 2003; Longnecker et al. 2002, 2007). None of these studies provided conclusive evidence that pre- or postnatal exposure were associated with negative effects on the male development.

### Benefits

Over the last decades, the benefits of breastfeeding for the infant and mother have been advocated extensively (Horta et al. 2007; James et al. 2009; WHO 2011). Thus, in any risk–benefit analysis the positive health effects of breastfeeding must be balanced against possible adverse health effects of these POPs. A large number of neonatal studies established significant positive health aspects of breastfeeding, but it is beyond the scope of our study to review all these studies in detail. In this review, we restrict ourselves to major outcomes and conclusions on this topic.

The most extensive and recent review on the positive health aspects of breastfeeding was done by the US Department of Health and Human Services, which included approximately 400 studies. It is concluded that breastfeeding reduces a large variety of health problems in early childhood, including the risk of acute otitis media, non-specific gastroenteritis, severe lower respiratory tract infections, atopic dermatitis, asthma (young children), obesity, possibly type 1 and 2 diabetes, childhood leukemia, SIDS and necrotizing enterocolitis (Ip et al. 2007). Quantifiable benefits of breastfeeding with respect to overall postnatal survival and hospitalization have also been reported, with a 30–40 % reduction in overall neonatal mortality and sudden infant death when breastfeeding. Furthermore, a longer breastfeeding period was associated with a decreasing risk in health problems (Chen and Rogan 2004). A study from Spain indicated that 30 % of the neonatal hospital admissions could be avoided for every additional month of breastfeeding (Paricio Talayero et al. 2006). Thus, there is compelling evidence that breastfeeding is associated with a reduced mortality and morbidity with many beneficial health effects for later life stages. In contrast, adverse health effects of POPs in human milk appear mostly transient in nature and less significant from a clinical point of view. The possible exception may be a reduced cognitive performance that can persist in later life (Ribas-Fito et al. 2006). In Table 2, a general overview is given for the observed benefits of breastfeeding for the infant and mother (Ip et al. 2007; James et al. 2009).

**Table 2:** General overview for the observed benefits of breastfeeding for the infant and mother (Ip et al. 2007; James et al. 2009)

*Benefits for the infant* Optimal nutrition Strong bonding with mother Safe milk Enhanced immune system Reduced risk of acute otitis media, gastroenteritis, lower respiratory tract infections and asthma Protection against allergies and intolerances Correct development of jaw and teeth Association with higher IQ/school performance Reduced risk of chronic diseases, e.g., obesity, diabetes, heart disease, hypertension, hypercholesterolemia, childhood leukemia Reduced risk of sudden infant death syndrome Reduced risk of overall morbidity and mortality	*Benefits for the mother* Strong bonding with infant Increased energy expenditure, faster return to prepregnancy weight Faster shrinking of the uterus Reduced postpartum bleeding and delay menstrual cycle Decreased risk of chronic diseases, e.g., breast and ovarian cancer, diabetes Improved bone density, decreased risk hip fracture Decreased risk postpartum depression Enhanced self-esteem in the maternal role Time and money saved from preparing and not buying formula, less medical expenses

## Conclusions and future perspectives

Based on our present knowledge, we conclude that the benefits of breastfeeding far outweigh the toxicological disadvantages that are associated with certain POPs. Subtle adverse health effects of DL compounds for the fetus and infant can still be expected from prenatal exposure due to current maternal body burdens that can still be found in many countries around the globe. Experimental and epidemiological studies indicate that future risk–benefit assessments should focus more on the in utero situation, rather than on the breastfeeding period. Consequently, the risk**–**benefit debate of breastfeeding may well be redundant. The results of our global monitoring study indicate that recent human exposure to DL compounds and PCBs is still above those considered toxicologically safe for the fetus and breastfed infant, while those for DDT would be acceptable for the countries studied. Our observations provide a strong argument to plea for further global source-directed measures to reduce human exposure further to dioxin-like compounds.
